# Eye Movements and the Intraorbital Subarachnoid Space: Potential Contribution of Altered Cerebrospinal Fluid Pumping in Optic Neuropathies

**DOI:** 10.1167/iovs.66.1.53

**Published:** 2025-01-23

**Authors:** Joseph L. Demer, Robert A. Clark, Soh Youn Suh, JoAnn A. Giaconi, Kouros Nouri-Mahdavi, Simon K. Law, Laura Bonelli, Anthony C. Arnold, Peter Quiros, Anne L. Coleman, Joseph Caprioli

**Affiliations:** 1 Department of Ophthalmology, University of California, Los Angeles, California, United States; 2 Stein Eye Institute, University of California, Los Angeles, California, United States; 3 Bioengineering Department, University of California, Los Angeles, California, United States; 4 Neuroscience Interdepartmental Program, University of California, Los Angeles, California, United States; 5 Department of Neurology, University of California, Los Angeles, California, United States

**Keywords:** anterior ischemic optic neuropathy (AION), cerebrospinal fluid (CSF), eye movement, glaucoma, optic nerve

## Abstract

**Purpose:**

The optic nerve (ON) is mechanically perturbed by eye movements that shift cerebrospinal fluid (CSF) within its surrounding dural sheath. This study compared changes in ON length and CSF volume within the intraorbital ON sheath caused by eye movements in healthy subjects and patients with optic neuropathies.

**Methods:**

Twenty-one healthy controls were compared with 11 patients having primary open angle glaucoma (POAG) at normal intraocular pressure (IOP), and 11 with chronic non-arteritic anterior ischemic optic neuropathy (NA-AION). High resolution magnetic resonance imaging (MRI) was performed in central and eccentric gazes, and analyzed to determine ON partial volume and gaze-related changes in ON path redundancy, ON elongation, and intrasheath CSF volume.

**Results:**

ON volume was subnormal in both POAG and NA-AION. In all subjects, ON path redundancy decreased similarly from abduction to central gaze to adduction; in healthy subjects, the ON path was also significantly less redundant in infraduction and supraduction. The ON elongated significantly in adduction in controls and NA-AION but not in POAG. In all groups, CSF volume was 40 to 50 mm^3^ in central gaze, and significantly decreased in adduction, abduction, and supraduction in controls but subnormally in adduction only in POAG and NA-AION. The globe translated laterally more than normal in NA-AION but did not retract.

**Conclusions:**

Horizontal gaze and supraduction change subarachnoid CSF volume around the retrobulbar ON. Eye movements might thus pump CSF to promote ON health, but this effect is subnormal in adduction in POAG and NA-AION, suggesting that retrobulbar CSF pumping is associated with chronic forms of these optic neuropathies.

The optic nerve (ON) is a mechanically dynamic part of the central nervous system (CNS): anteriorly, the ON's attachment to the globe experiences large and numerous rotational perturbations, including over 180,000 saccades daily.^1^ The intraorbital ON is surrounded and protected by a sheath of dura mater contiguous with sclera anteriorly, and periosteum of the bony optic canal rigidly posteriorly. Within the ON sheath, cerebrospinal fluid (CSF) is present among arachnoid trabeculations that extend from the inner dural surface to the pial outer surface of the ON^2^ that is contiguous with a dense intercalating network of collagenous supporting tissue within the internal substance of the ON.^3^ Important physiological roles have been implicated for the CSF in CNS health, including waste removal, as has been extensively reviewed.^4^ The CSF that bathes the retrobulbar ON is contiguous with the CSF that fills the ventricles and surrounds the brain and spinal cord. The total CSF volume is 124 to 150 mL and it is recycled 3 to 5 times daily.^5^ However, the ON head at the posterior eye is at the end of a long and narrow cul-de-sac in the CSF system,^5^ which would seem problematic for CSF flow to the eye. Whereas glymphatics have been proposed as a possible outflow pathway for retrobulbar CSF,^6^ their quantitative contribution is unknown.

Driving forces for CSF flow have classically been speculated to include CSF absorption in the arachnoid granulations, ventricular pulsations, and vascular pulse pressure in the choroid plexus, intermittently augmented by CSF pressure spikes during postural changes and Valsalva maneuvers.^7^^,^^8^ Moreover, disturbance of CSF flow around the ON has been proposed to be capable of causing a pathological compartment syndrome.^9^ Evidence for a compartmental syndrome has been suggested in patients with primary open angle glaucoma (POAG) and normal intraocular pressure (IOP) in whom computer tomography-assisted cisternography demonstrated progressively diminishing contrast concentration in an enlarged retrobulbar subarachnoid space,^10^ and diffusion weighted magnetic resonance images (MRIs) showing reduced CSF flow-range ratio between the intracranial cavity and the subarachnoid space of the ON.^11^ It has been speculated that POAG without elevated IOP, a condition also known as normal tension glaucoma (NTG), may be caused by age-related reduction in CSF turnover with diminished clearance of toxic substances,^12^ and specifically a disorder of CSF glymphatic flow.^13^^–^^15^

Scant attention has so far been directed to the possible role of eye movements as possible drivers of CSF flow in the retrobulbar subarachnoid space. There have been many overlooked clues. The retrobulbar ON has been noted in MRI to shift within its dural sheath during horizontal ductions,^16^ and physiologic large adduction straightens and stretches the ON and sheath.^17^ Average ON length in healthy subjects is insufficient to allow unhindered rotation, tethering the globe in adduction^17^ exceeding about 26 degrees.^18^ Consequently, the stretched ON and its sheath induce gaze direction-related traction on the optic disc and peripapillary region, as confirmed by optical coherence tomography (OCT) in living subjects.^19^^,^^20^ Relative displacements^21^ and tilting^22^ of the optic disc during horizontal duction have been demonstated by OCT. Local area changes^23^ and shear strains^24^ in the optic disc, as well as relative displacements^25^ and strains^26^ of retinal blood vessels, also occur during horizontal eye rotation. Moreover, the healthy ON and its sheath stretch during adduction tethering,^27^ but in POAG the ON does not similarly stretch.^16^^,^^28^ All of these deformations in and around the retrobulbar subarachnoid space (SAS) might contribute to CSF displacement or flow.

By supplementing high resolution MRI with technical measures to suppress eye movements during scanning, it is possible to quantitate the SAS surrounding the intraorbital ON. The current study addressed the question of changes in retrobulbar subarachnoid CSF using high resolution MRI to compare the effect of horizontal and vertical eye rotations on SAS in healthy subjects and subjects with POAG despite normal IOP. In view of the known role of ON tethering in adduction, horizontal ductions were emphasized, but exploratory comparisons were also made of vertical ductions in normal subjects and in subjects with a history of non-arteritic anterior ischemic optic neuropathy (NA-AION). The pathology of NA-AION remains mysterious, as recently reviewed in detail.^29^ The effects of vertical ductions on the normal ON have not been previously described.

## Methods

This study analyzed orbital MRI collected per prospective protocols approved by the Institutional Review Board for Protection of Human Subjects of the University of California, Los Angeles, and conformed to the tenets of the Declaration of Helsinki. All subjects gave written informed consent prior to participation.

A group of 11 subjects with POAG without elevated IOP were under the care of study authors who are glaucoma specialists, and were diagnosed based upon OCT evidence of retinal nerve fiber layer loss correlating with glaucomatous visual field defects on automated perimetry, accompanied by characteristic optic disc features.^30^ Records of subjects with POAG included IOP measurements on multiple days, ON photos, ON imaging with OCT, and achromatic perimetry (with Humphrey Field Analyzer programs 24-2 or 30-2 SITA standard strategy). None of these subjects had been observed to have IOP elevated above the statistical normal maximum of 21 millimeters of mercury (mm Hg). These subjects were included in a previous study of the effects of horizontal duction in glaucoma.^28^ Subjects were excluded from this group if they had previously undergone intraocular surgeries besides those for cataract, glaucoma, or refractive error.

A group of 11 subjects with a history of NA-AION in one or both eyes were recruited over a period of 5 years from the practices of study authors who are neuro-ophthalmologists. Diagnosis was based on a history of acute, painless monocular visual loss, characteristic optic disc appearance with corresponding visual field defects, and absence of evidence for alternative pathologies, such as glaucoma or intracranial mass. Subjects were excluded from this group if they had laboratory or examination evidence of arteritis, had previously undergone intraocular surgeries besides those for cataract, glaucoma, or refractive error, or if they had any other cause for optic neuropathy besides NA-AION. All subjects with NA-AION were in the subacute to chronic phase at least 40 days following onset of acute visual loss; all optic discs appeared at this time atrophic rather than edematous at the time of enrollment.

A control group of 21 subjects was recruited by advertisement. Some of these subjects had participated in the earlier MRI study of horizontal duction^28^; additional subjects were recruited for MRI that included vertical and horizontal ductions. Control subjects were required to have best corrected visual acuity of 20/20 in each eye, normal IOP, and no history of ocular surgeries besides those for cataract or refractive error, no ocular trauma, and no other ocular disorder except for refractive error or lens opacity. An age-matched subgroup of 16 controls was isolated by sequentially excluding the youngest subjects until the average age approximated the POAG and NA-AION groups.

### MRI Acquisition

**Figure 1.**
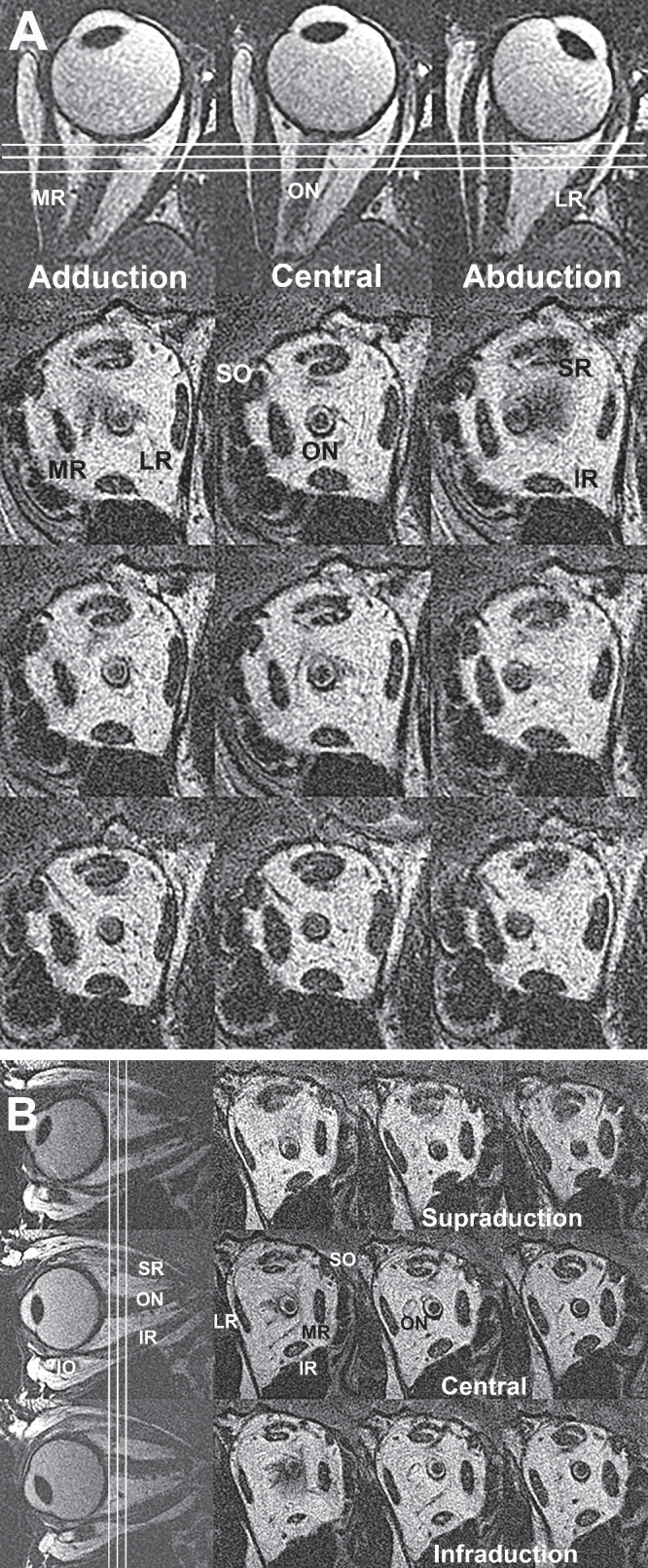
(**A**) Horizontal duction imaging. Axial T2 MRI (*top row*) with superimposed *white lines* designating the contiguous 2 mm thick quasi-coronal planes illustrated sequentially in the lower 3 rows. IR, inferior rectus muscle; LR, lateral rectus muscle; MR, inferior rectus muscle; ON, optic nerve; SO, superior oblique muscle; SR, superior rectus muscle; (**B**) Vertical duction imaging. Quasi-sagittal T2 MRI with superimposed white lines designationg the contiguous 2-mm thick quasi-coronal planes illustrated in the right 3 rows and columns. IO, inferior oblique muscle.

The first author personally performed high resolution MRI using a 1.5T General Electric Signa scanner and surface coils (Medical Advances, Milwaukee, WI, USA), or a 3.0T General Electric Symphony Scanner and surface coils (Resonance Innovations, Omaha, Nebraska, USA) using a T2 fast spin echo pulse sequence, as previously described.^16^^,^^31^^–^^35^ Eye position was controlled by monocular fixation of a fiberoptic illuminated target located in central position, or laterally to establish abduction of the fixating eye with simultaneous adduction of its fellow because the surface coil occludes the adducting eye. Vertically eccentric targets were presented to the scanned eye. Axial 2-mm thick images (10–12 cm field of view, 256 × 256 matrix) including both orbits were obtained to determine horizontal gaze direction. Quasi-coronal sets of 17 to 20, 2-mm thick planes perpendicular to the long axis of each orbit were obtained separately (field of view 8 × 8 cm, 256 × 256 matrix, resolution 312 microns). For all subjects, acquisitions were repeated for central gaze and abduction and adduction of each eye (Fig. 1), for which limited results were included in our earlier publication.^16^ For the additional control subjects and subjects with NA-AION, MRI was also repeated during infraduction and supraduction. Vertical gaze direction was determined from quasi-sagittal sets of planes parallel to the long axis of each obtained separately for each orbit (field of view 8 × 8 cm, 256 × 256 matrix, resolution 312 µm). Quasi-coronal sets of 17 to 20, 2-mm thick planes perpendicular to the long axis of each orbit were then obtained separately for supraduction and infraduction.

The MRI analysis was performed by the same investigator RAC, as elsewhere described,^16^ using ImageJ 64 and custom software. In representative subjects in each group, axial (see Fig. 1A) or sagittal images (see Fig. 1B) were obtained; typical gaze direction was computed from these images as the angle of a line connecting the lens plane perpendicular to the fovea.^36^ Such gaze direction images were not obtained in every subject in order to minimize scanning time, but were obtained for typical target locations used for all subjects. Gaze direction determination by MRI does not account for differences between anatomic and subjective visual directions, so measured changes in anatomic angular eye position^36^ are not equivalent to subjective gaze angles reported in OCT studies.^18^^,^^37^

All other analyses were performed in quasi-coronal images that were obtained in every subject, because this provided maximum overall resolution for the SAS. In each 2-mm thick image plane, the ON and external border of the bright subarachnoid CSF were separately outlined manually using a digital cursor. Next followed automated calculation of horizontal and vertical coordinates of the ON area centroid, and the third dimension corresponding to the image plane's anteroposterior coordinate.^16^ Cross sectional area of the ON was automatically calculated, and in all image planes where subarachnoid CSF was distinguishable, subtracted from the cross sectional area of the external border of the CSF to obtain the SAS cross-sectional area. Path length and straightness of the ON were obtained by analysis of quasi-coronal MRI. Cartesian distances between centroids were summed to compute overall path lengths. Minimum geometric distances between the globe-ON junction and the most posterior image plane in which the ON was visible were computed. The ratio between actual and minimum distance from the globe and the most posterior point imaged, was taken as a measure of straightness.^16^

Analysis of the ON volume and CSF volume in the SAS was as previously published, and is briefly summarized here.^27^ The most anterior image plane including both the ON and globe contained the globe-ON junction. Three-dimensional distances between ON area centroids in adjacent planes were summed to calculate the ON path length, whereas angular differences between area centroids were used to correct cross-sectional areas for obliquity.^38^ Cross-sections of the ON in adjacent image pairs were averaged to compute interslice ON cross-sectional area for that image pair. Changes in ON interslice cross-sectional areas were compared between gaze positions to determine elongation or compression. Change in ON length for each interslice pair was given by initial divided by final ON cross-section, multiplied by initial ON interslice length. This calculation was repeated for all interslice regions, and the differences in ON lengths summed to compute total gaze-related ON elongation. Volume of the SAS for each interslice segment was calculated by subtracting the ON cross-sectional areas from the areas bounded by the inner border of the ON sheath. The SAS cross-sectional areas in adjacent image slices were averaged and multiplied by ON length between those two image slices, corrected for path obliquity, to calculate the SAS partial volume (PV) for that ON segment. Segmental changes were summed for all four interslice regions to compute the total SAS PV change.

### Statistics

Parametric statistical analyses were performed using GraphPad Prism software (GraphPad Software, LaJolla, CA, USA) with the individual eye as the unit of analysis, but confirmed using generalized estimating equations (GEE) in SPSS software (IBM Corporation, Armonk, NY, USA) that corrects for possible confounding by possible interocular correlations between eyes of individual subjects.^39^ Because there has been no prior study of intraorbital SAS volume in humans, sample size for this measure could not be estimated a priori. However, based on measurement variance in our published MRI data,^28^ a sample consisting of both eyes of 10 subjects per group would be required to detect with 90% power the observed 0.5 mm difference in globe retraction in adduction observed between Caucasian subjects with POAG without elevated IOP versus controls. All groups exceeded 10 subjects each in this study.

## Results

The control group consisted of 6 men and 15 women, with average age 53 ± 19 years (standard deviation [SD], range 21 to 71 years). By elimination of the youngest subjects, an age-matched subgroup was selected consisting of 4 men and 12 women, with average age 61 ± 11 years (range 44 to 71 years). The group with POAG consisted of 5 men and 6 women with average age 63 ± 11 years (range 48 to 78 years). Mean deviation on 24-2 visual field examination averaged –6.5 ± 5.8 dB in subjects with POAG in whom vision was sufficient for quantitative testing.

The group with NA-AION consisted of 6 men and 5 women, of average age 60 ± 13 years (range 43 to 70 years). Three subjects were bilaterally, sequentially affected, and 8 unilaterally affected. Mean perimetric deviation in affected eyes was –16 ± 9.4 dB, and –1.1 ± 2.5 dB in unaffected eyes. The minimum interval from onset of NA-AION to imaging was 40 days.

**Figure 2.**
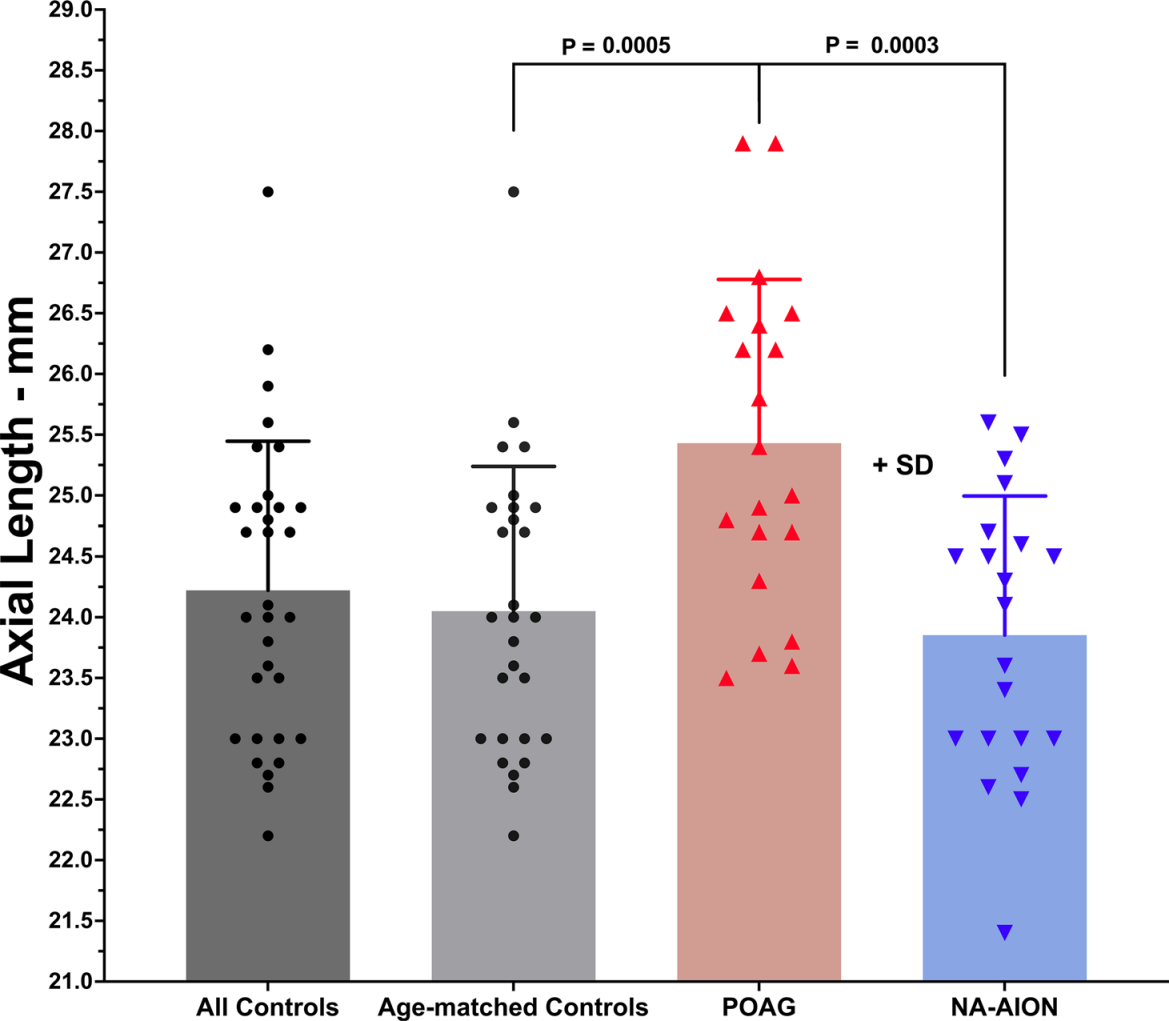
Mean axial length of distance from the anterior corneal surface to the retina did not differ significantly from controls in non-arteritic anterior ischemic optic neuropathy (NA-AION), but was significantly greater in primary open angle glaucoma (POAG). SD, standard deviation. *P* values by Student *t*-testing. Each symbol represents one eye.

Axial length was determined from axial MRI as the maximum distance from the anterior corneal surface to the retina and is plotted in Figure 2. Axial length was 24.2 ± 1.3 mm in the full control group, 24.0 ± 1.2 mm in age-matched controls, and 23.9 ± 1.1 mm in subjects with NA-AION. None of these values differed significantly from one another, but axial length in subjects with POAG was significantly greater than NA-AION and controls at 25.4 ± 1.3 mm (*P* < 0.005).

### Duction Angles

**Figure 3.**
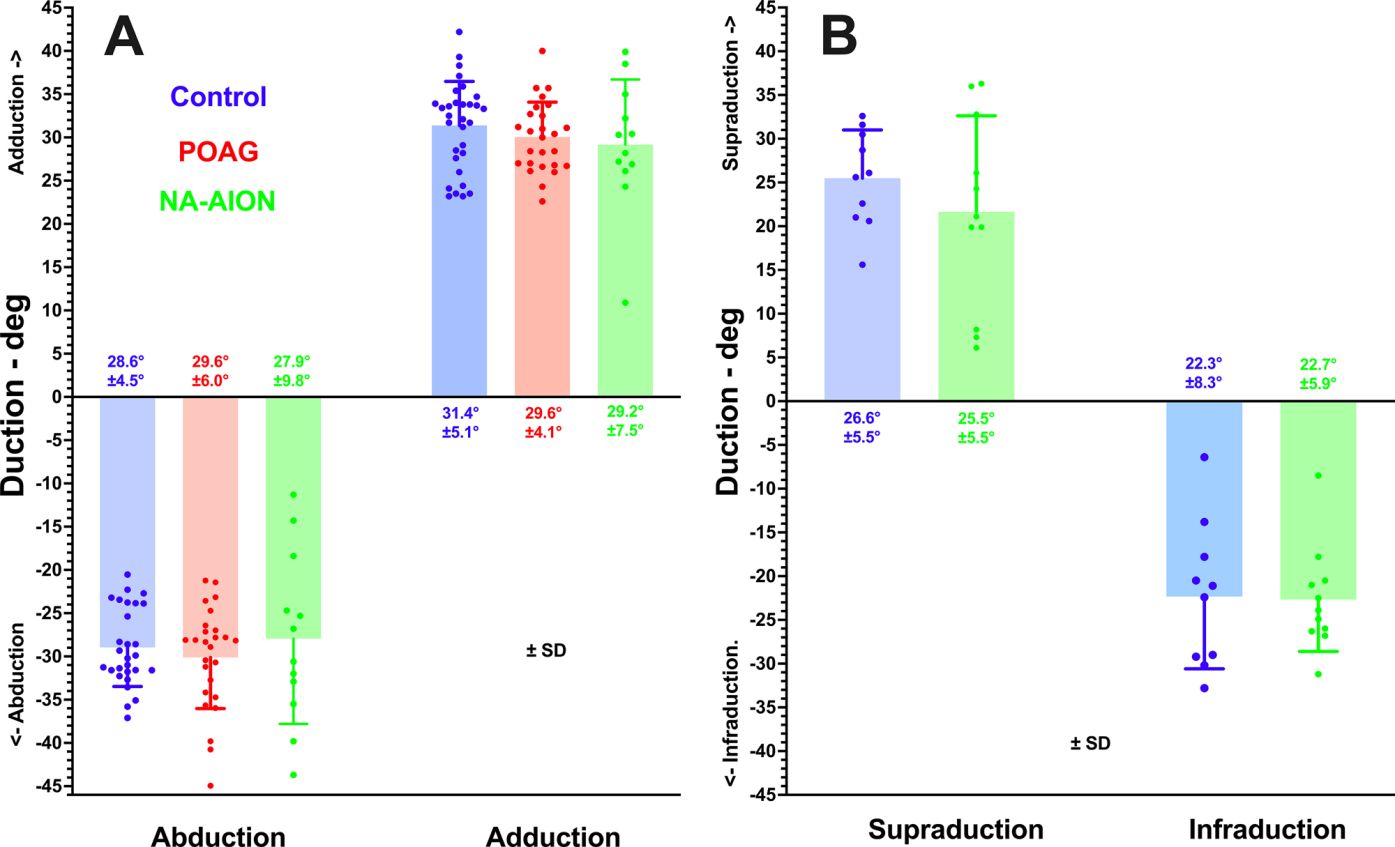
Mean horizontal and vertical duction angles ± standard deviation (SD) achieved in the subject groups. Horizontal ductions (**A**) 38 eyes of 21 control subjects, 20 eyes of 11 subjects with POAG, and 18 eyes of 11 subjects with NA-AION. Vertical ductions (**B**) 10 eyes of 6 control subjects, and 11 eyes of 6 subjects with NA-AION. Each symbol represents one eye.

In the interest of minimizing scanning time, duction angles were measured only in representative samples of the three subject groups (Fig. 3). These measurements were obtained for horizontal ductions in 38 eyes of 21 control subjects, 20 eyes of 11 subjects with POAG, and 18 eyes of 11 subjects with NA-AION. In all groups, abduction averaged 28 degrees to 30 degrees, and adduction 29 degrees to 31 degrees. Vertical duction was imaged only in controls and NA-AION. Measurements were made for vertical ductions for 10 eyes of 6 control subjects, and 11 eyes of 6 subjects with NA-AION. Supraduction averaged 25 degrees to 27 degrees, and infraduction was 22 degrees to 23 degrees.

### Subacute Optic Nerve Findings in NA-AION

**Figure 4.**
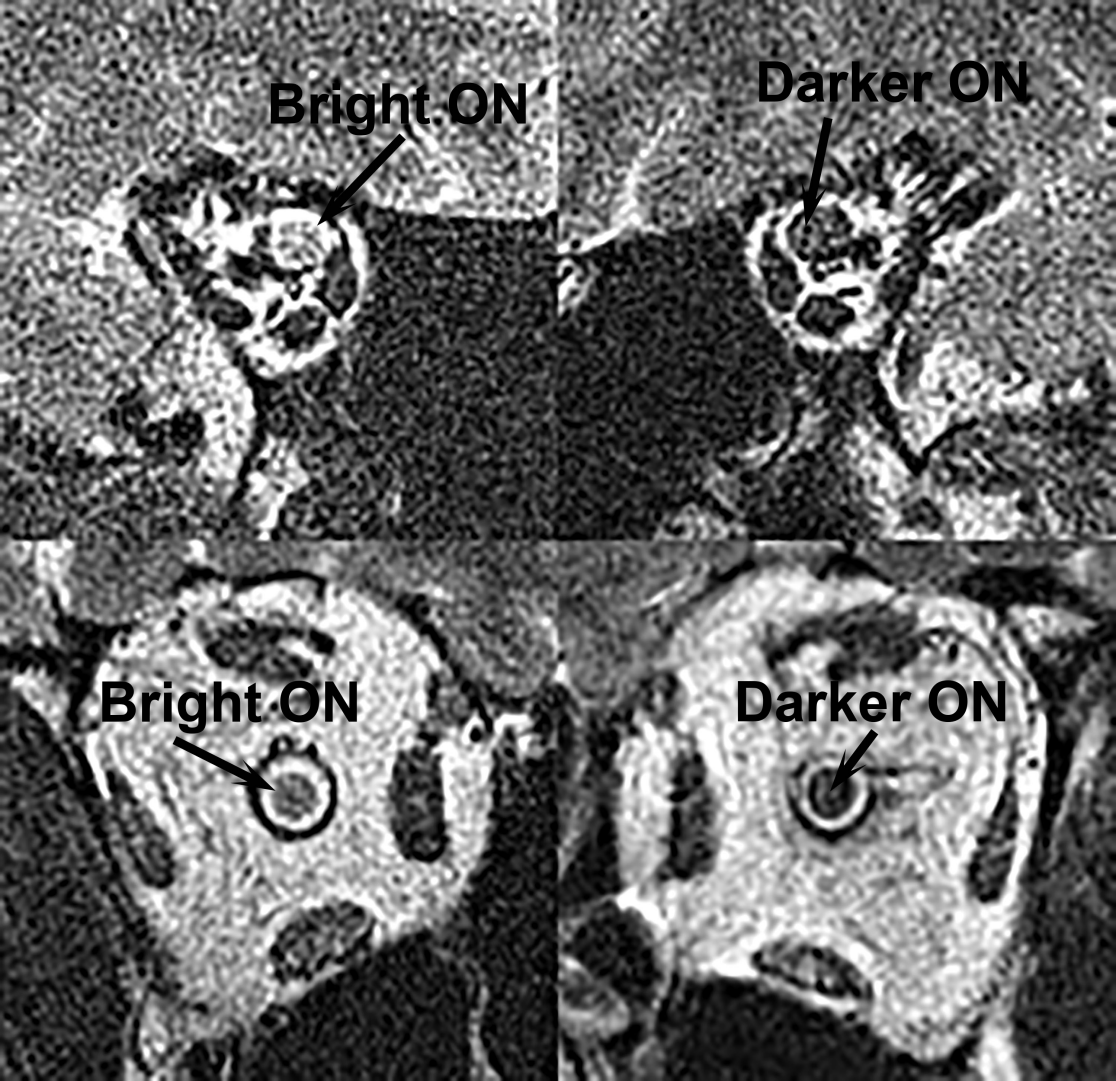
Subacute bright signal in the optic nerve (ON) in NA-AION. Quasi-coronal MRI of the right and left orbits of a 64-year-old woman imaged 42 days following acute onset of NA-AION in the right eye.

Two subjects with relatively recent onset NA-AION exhibited longitudinally extensive T2 bright signal in the affected ON. Identical imaging parameters were used for both orbits of each subject, and brightnesses for all nearby tissues were comparable, so this finding is not attributable to variations in the imaging technique. The bright signal in the affected ON is clearly shown in Figure 4 for a 64 year-old woman imaged 42 days following acute onset of NA-AION in the right eye that reduced central acuity to 20/25 with afferent pupillary defect, dyschromatopsia, and arcute visual field defect with a –13.5 dB visual field mean deviation. Similar findings were evident in a 65-year-old man approximately 90 days following onset of NA-AION, but were not demonstrable in a 46-year-old man 135 days following onset, nor in a 66-year-old woman at least several months following onset. The remaining three other subjects with unilateral NA-AION could not be evaluated for this finding due to technical limitations on imaging, usually due to poor fixation resulting from central scotomata in the involved eyes.

**Figure 5.**
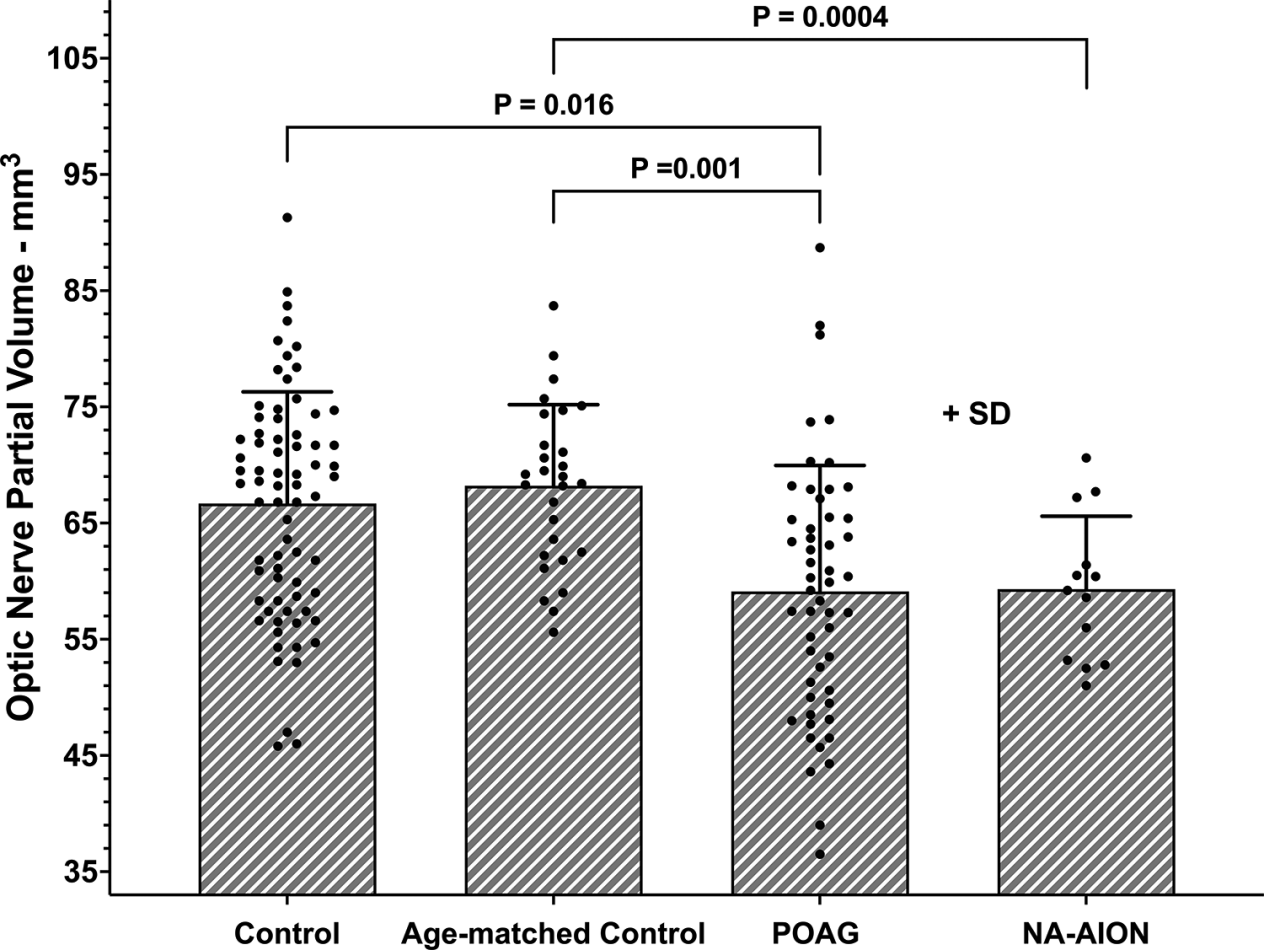
Mean optic nerve partial volume. Error bars indicate standard deviation. *P* values are based on Student *t*-testing. Indicative of optic neuropathy, volume was significantly subnormal in POAG and NA-AION. Each symbol represents one eye. SD, one standard deviation.

Optic nerve partial volume averaged about 65 mm^3^ for the control groups in the analysis region and is plotted for all groups in Figure 5. Compared with age-matched controls at 68.2 ± 7.0 mm^3^ (µL), average ON partial volume was significantly lower in POAG at 61.4 ± 12.4 mm^3^ (*P* < 0.001), and in NA-AION at 60.0 ± 6.5 mm^3^ (*P* < 0.004). This difference represents an approximately 10% reduction relative to age-matched controls for both subject groups. Including both NA-AION and POAG (considered always bilateral), 1-way ANOVA indicated that ON partial volume was significantly smaller in affected than unaffected eyes (*P* = 0.009).

### Optic Nerve Path Redundancy

**Figure 6.**
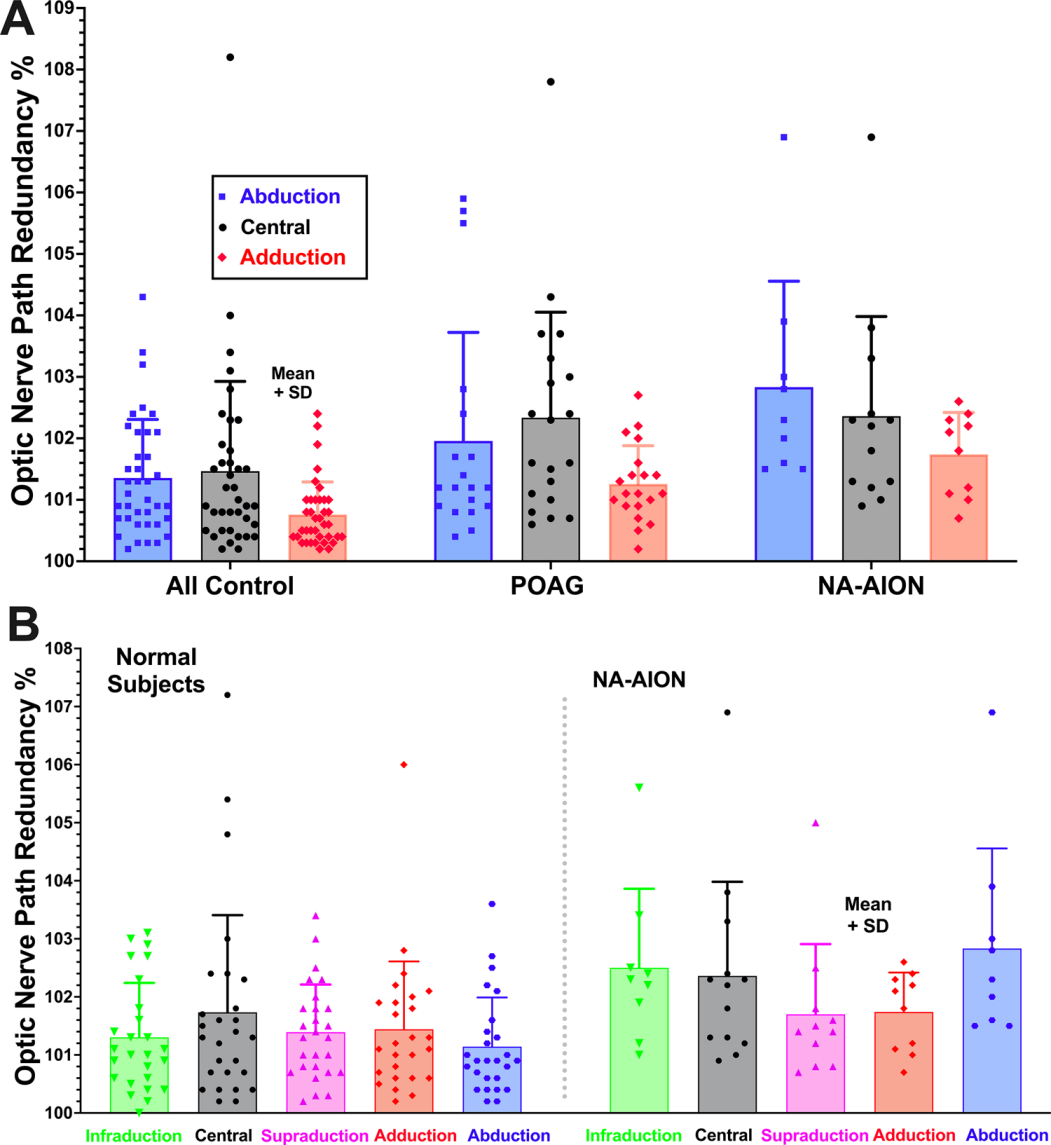
Mean optic nerve path redundancy in multiple gaze directions. (**A**) Effect of horizontal gaze was significant by ANOVA at *P* < 0.001 for all groups, but GEE analysis did not indicate a significant effect of diagnosis. (**B**) Redundancy in NA-AION was significantly greater than normal by ANOVA at *P* < 0.001, but there was no significant effect of gaze direction. POAG, primary open angle glaucoma; NA-AION, non-arteritic anterior ischemic optic neuropathy; SD, one standard deviation. Each symbol represents one eye.

In both POAG and NA-AION, average ON path redundancy in central gaze was identical at 102.3%, which was insignificantly greater than for controls. In adduction, ON path redundancy in controls and POAG was significantly less than in central gaze (*P* < 0.02), but this trend in NA-AION was not significant (*P* > 0.15; Fig. 6A). For the smaller number of subjects scanned in both horizontal and vertical gazes, ANOVA did not demonstrate a significant effect of gaze direction but did demonstrate significantly greater redundancy in NA-AION than healthy controls(*P* < 0.001; Fig. 6B).

### Optic Nerve Elongation

**Figure 7.**
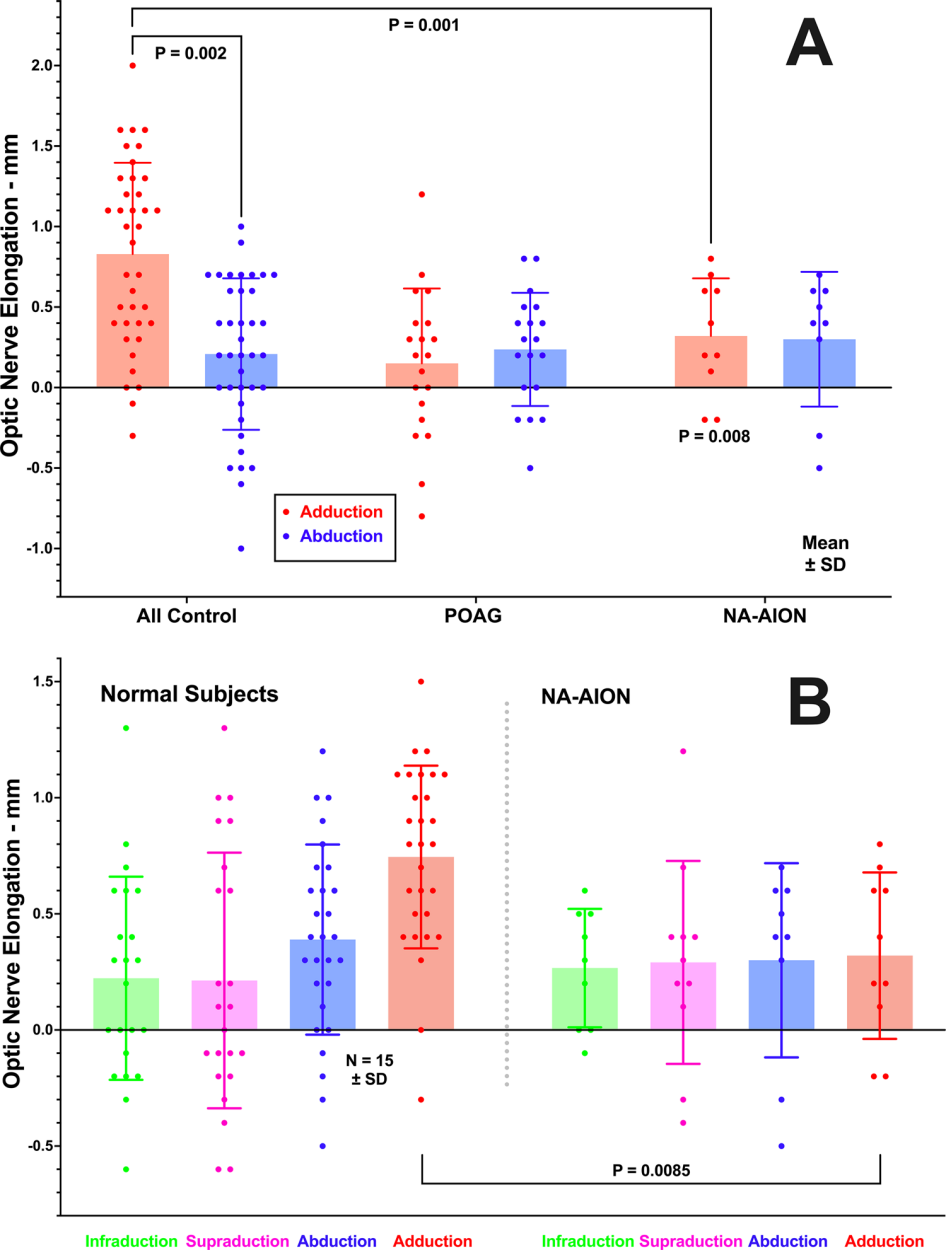
Mean optic nerve elongation from its central gaze length in 38 orbits of 21 control subjects, 21 orbits of 11 subjects with non-arteritic anterior ischemic optic neuropathy (NA-AION), and 20 orbits of 11 subjects with primary open angle glaucoma (POAG). (**A**) Ab- and adduction, demonstrating significant elongation in adduction, but greater in control subjects than in NA-AION, where elongation was nevertheless significantly greater than zero (*P* = 0.008). Elongation was not significant in adduction in POAG. Elongation in abduction occurred only in POAG (*P* = 0.007). (**B**) In NA-AION, there was no significant ON elongation in any horizontal or vertical gaze direction. Each symbol represents one eye. SD, one standard deviation.

In adduction, the ON elongated significantly by 0.83 ± 0.56 mm in 38 orbits of 21 healthy control subjects (*P* < 0.0001 vs. zero); this elongation was significantly greater than in 21 orbits of 11 subjects with NA-AION, although in that group, elongation was also significantly greater than zero at 0.29 ± 0.40 mm (*P* = 0.008; Fig. 7). The ON did not elongate in adduction in 20 orbits of 11 subjects with POAG (*P* = 0.18). There was generally no significant ON elongation in abduction in any group, except for 0.23 ± 0.33 mm in the group with POAG (*P* = 0.007). Moreover, in both controls and in NA-AION, there was no significant ON elongation in abduction, infraduction, or supraduction (see Fig. 7B).

### Anteroposterior and Lateral Globe Positions

**Figure 8.**
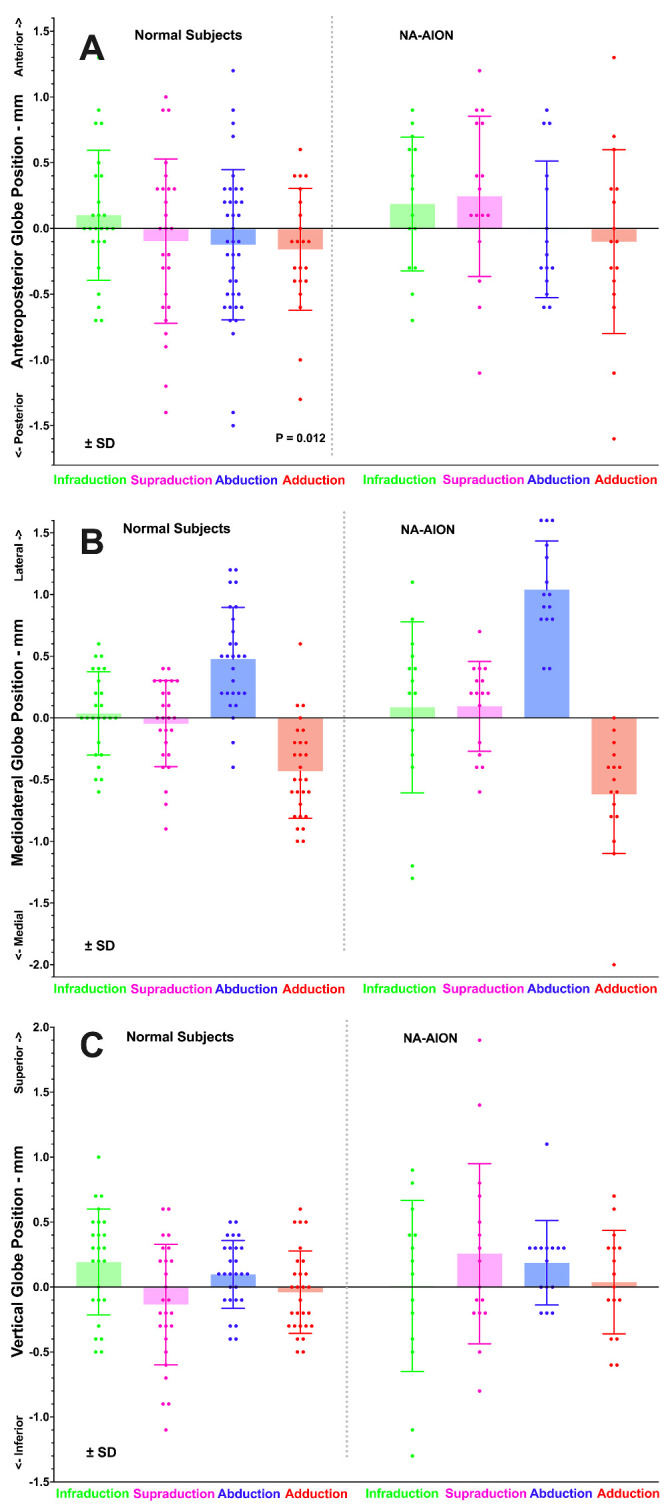
Mean globe translation during duction in 14 healthy subjects and 11 subjects with non-arteritic anterior ischemic optic neuropathy (NA-AION). (**A**) Except for slight posterior translation in adduction in normal subjects, there was no appreciable anteroposterior transation during vertical or horizontal duction. Differences were insignificant by ANOVA. (**B**) In both groups there was significant lateral translation in abduction. (**C**) There was no significant vertical translation in either group. Each symbol represents one eye.

In subjects with NA-AION and in control subjects, the anteroposterior position of the globe was not significantly different in horizontal or vertical eccentric gazes from that in central gaze (*P* > 0.13; Fig. 8A). This finding indicates absence of globe retraction in eccentric gaze. Mediolateral translation in vertical gaze was also absent in both subject groups (Fig. 8B). However, both groups demonstrated highly significant lateral globe translation in abduction, averaging 0.48 ± 0.42 mm in controls and significantly greater at 1.03 ± 0.38 mm in subjects with NA-AION (*P* < 0.001). Both groups demonstrated similar nasal translation in adduction: 0.43 ± 0.37 mm in controls and 0.62 ± 0.47 mm in NA-AION (*P* = 0.18). Globe translation in POAG is not presented here, because it has been published in detail elsewhere.^16^^,^^28^

**Figure 9.**
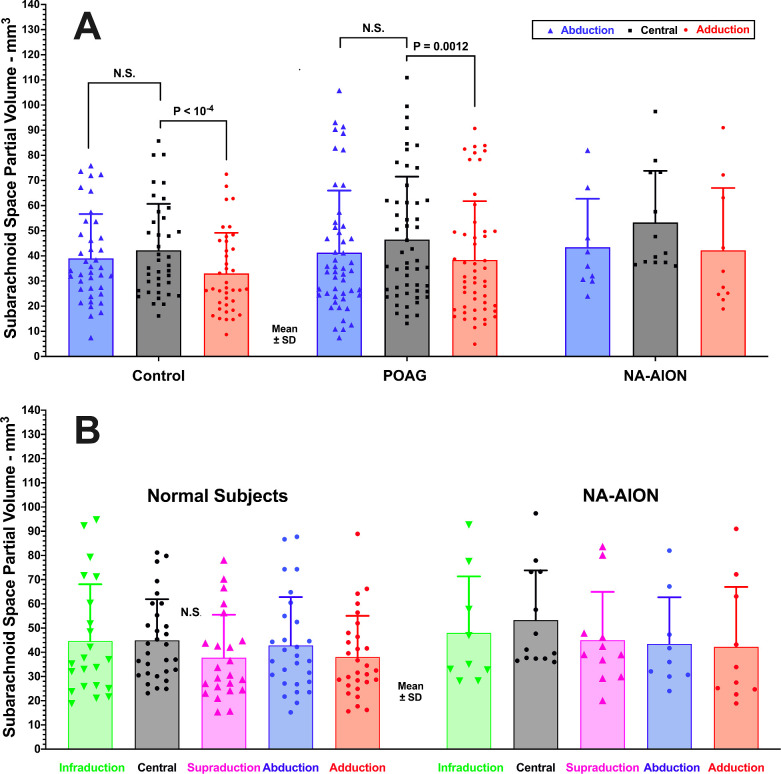
Subarachnoid space partial volume. (**A**) Central gaze and horizontal duction in control subjects, subjects with primary open angle glaucoma (POAG), and non-arteritic anterior ischemic optic neuropathy (NA-AION). Partial volume was significantly lower in adduction than in central gaze in controls and POAG, but not in NA-AION despite a similar numerical trend. In abduction, partial volume was not significantly different from central gaze in any subject group. (**B**) Partial volume did not change significantly in supraduction or infraduction in healthy subjects or subjects with NA-AION. Each symbol represents one eye.

The subarachnoid space volume was approximately 40 to 55 mm^3^ in central gaze in healthy subjects (whether or not they were age-matched), and was not significantly different in POAG or NA-AION (*P* > 0.1; Fig. 9). Averaging over all subjects in all groups, for women, SAS volume in central gaze was 39.9 ± 18.8 mm^3^, but for men it was significantly greater at 55.7 ± 24.5 mm^3^ (*P* = 0.002 by 1-way ANOVA). In affected eyes with NA-AION, SAS volume in central gaze was marginally greater at 57.3 ± 25.4 mm^3^ than 44.9 ± 16.6 mm^3^ in unaffected eyes (*P* = 0.07 *t*-test); this difference was significant by GEE analysis (*P* = 0.003).

In healthy subjects, SAS volume decreased significantly from 42.7 ± 18.5 mm^3^ in central gaze to 32.3 ± 15.6 mm^3^ in adduction (*P* < 0.0001), and in POAG from 45.3 ± 27.3 mm^3^ in central gaze to 37.3 ± 24.8 mm^3^ in adduction (*P* = 0.0045). In NA-AION, SAS volume in central gaze was 52.9 ± 23.0 mm^3^, but not significantly different in adduction at 38.3 ± 21.2 mm^3^ (*P* = 0.062); however, because SAS volume could not be measured in adduction in 8 orbits with NA-AION, the lack of significance despite the same magnitude of change may be due to small sample size.

**Figure 10.**
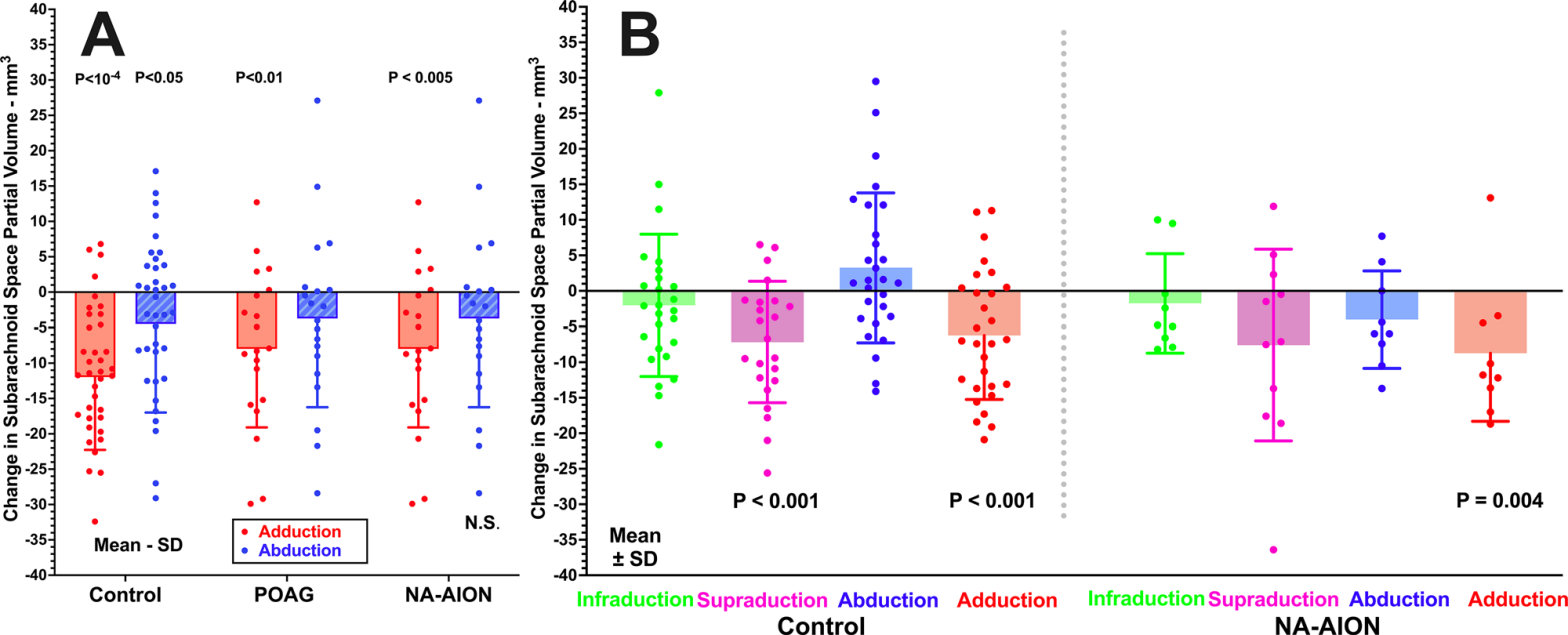
Gaze-related change in subarachnoid space (SAS) partial volume. (**A**) Both ab- and adduction significantly reduced volume in controls, but only adduction did so in subjects with primary open angle glaucoma (POAG) and non-arteritic anterior ischemic optic neuropathy (NA-AION). (**B**) Supraduction and abduction significantly reduced (SAS) partial volume in control subjects, but and adduction did so in NA-AION. Each symbol represents one eye. *P* values are based on Student *t*-test. The significance levels are for comparison with zero, and are not significant when not listed.

The change in subarachnoid space volume was computed as the difference for each eye between eccentric and central gazes and is shown in Figure 9. In healthy subjects, both ad- and supraduction reduced average SAS partial volume. Adduction reduced SAS volume of the entire control group by 12.8 ± 11.6 mm^3^ (*P* < 0.0001), which ANOVA showed to be significantly greater than the significantly non-zero values of 8.0 ± 11.1 mm^3^ in POAG and 7.7 ± 9.1 mm^3^ in NA-AION (*P* < 0.001; see Fig. 9A). In healthy control subjects, adduction also significantly reduced SAS volume by 4.5 ± 12.5 mm^3^ (*P* = 0.01), whereas the reductions were insignificant in POAG and NA-AION. In subjects with POAG, adduction significantly reduced SAS partial volume by 8.0 ± 11.1 mm^3^ (*P* < 0.004), whereas abduction did not (see Fig. 9A). Pooling all subject groups, gender had no effect on the change in SAS partial volume due to horizontal gaze ab- or adduction. Supraduction significantly reduced SAS volume in controls by about 7 mm^3^ (Fig. 10B). Gaze-related changes in SAS volume did not differ significantly among subject groups by *t*-testing.

### Affected Versus Unaffected Nerves in NA-AION

Partial volume in central gaze of the 8 affected ONs in unilateral NA-AION was 60.7 ± 7.2 mm^3^, slightly but not significantly smaller than the contralateral unaffected ONs at 63.0 ± 9.5 mm^3^ (*P* = 0.5). None of the other measures evaluated in subjects with unilateral NA-AION differed significantly between affected and unaffected eyes.

### Generalized Estimating Equation Analysis

All measures of specific interest, or statistically significant by parametric statistical testing that assumes independence of right and left eyes within subjects, were also analyzed using GEE that accounts for possible inter-eye correlations. Subject age and axial length were used as covariates and gender as a factor, in these analyses. Partial volume of the ON was confirmed by GEE to be significantly less than healthy subjects for both POAG and NA-AION after controlling for age (*P* < 0.001 for both), although age and axial length also had significant effects (*P* < 0.001); gender did not have a significant effect. By GEE, ON path redundancy in central gaze was not significantly related to gender or age, but was independently related both to diagnosis and to axial length (*P* = 0.001). Elongation of the ON in adduction was significantly related to diagnosis (*P* < 0.001) and axial length (*P* = 0.002) but not age or gender, nor to affected versus unaffected eye status in NA-AION. Comparing the full control group with POAG and NA-AION, GEE demonstrated a significant effect of axial length (*P* = 0.046) and diagnosis (*P* = 0.005) but not gender on mediolateral globe translation in adduction, and axial length only in abduction (*P* < 0.001). By GEE, volume of the SAS in central gaze was not significantly related to diagnosis of POAG or NA-AION, or to axial length, but was related to gender (*P* = 0.012), confirming the result of ANOVA. By GEE the SAS volume in central gaze in subjects with NA-AION was significantly less at 44.9 ± 16.6 mm^3^ in unaffected than in affected eyes at 57.3 ± 25.4 mm^3^ (*P* = 0.003), although this difference was not significant by *t*-testing (*P* = 0.07). The change in SAS partial volume during adduction (Fig. 10A) was demonstrated by GEE to be highly significantly greater in controls than in both POAG and NA-AION (*P* < 0.001). Thus, the inferences of statistical significance indicated by *t*-testing were confirmed and some extended by GEE, demonstrating absence of exaggeration of statistical significance by intereye correlations within individual subjects.

## Discussion

### Optic Nerve Changes

High resolution MRI demonstrated the novel but unsurprising finding in NA-AION of approximately 8 mm^3^ (about 10%) reduction in partial volume of the intraorbital ON in the region of analysis extending 10 mm posterior to the globe. This reduction is quantitatively similar to the subjects with POAG without elevated IOP and consistent with ON atrophy in both NA-AION and POAG as well as postmortem measurements in POAG.^40^ In NA-AION, ON partial volume in affected eyes was about 8 mm^3^ less in affected than unaffected eyes, which is roughly similar to the 12 mm^3^ greater SAS volume in affected eyes, suggesting that CSF approximately replaced the void left by ON atrophy in unilateral NA-AION. Volume of the ON was unrelated to subject gender, yet, for women, SAS volume in central gaze was significantly less at 39.9 ± 18.8 mm^3^ than 55.7 ± 24.5 mm^3^ for men (*P* = 0.002).

While the current MRI confirms ON path straightening in adduction in normal subjects and subjects with POAG, subjects with NA-AION exhibited shortening of the ON path in adduction. In healthy subjects, the straight ON stretched in adduction (see Fig. 7) without producing globe retraction, whereas in POAG the ON did not stretch in adduction (see Fig. 7) and the globe retracted. This contrasts with NA-AION, in which there was only slight ON elongation in adduction (see Fig. 7A) without globe retraction, presumably because the ON was too redundant to create a tethering phenomenon in adduction. In none of the subject groups studied did the ON elongate in infraduction or supraduction, nor was vertical duction associated with globe retraction.

Volume of the CSF in the SAS in the anterior retrobulbar region in central gaze demonstrated an unexpected gender dimorphism, being significantly less for women at 39.9 ± 18.8 mm^3^ than for men at 55.7 ± 24.5 mm^3^ (*P* = 0.002). The possible importance of this difference is not evident. Volume in the SAS was similar to normal controls in POAG and NA-AION, albeit greater in affected than unaffected eyes in the latter as likely reflective of optic atorphy. Abduction, adduction, and supraduction all decreased SAS volume in this region by 6 to 12 mm^3^ (µL) in all subject groups. In particular, the change in SAS volume in adduction of 12.8 ± 11.6 mm^3^ in healthy controls was significantly reduced by about 30% in both optic neuropathies, to 8.0 ± 11.1 mm^3^ in POAG and 7.7 ± 9.1 mm^3^ in NA-AION (*P* < 0.001; see Fig. 9A). These findings suggest that gaze-related changes in SAS volume may be related to these disease processes.

### Eye Movements as a CSF Pump

The ubiquity of gaze-related changes in SAS volume should not motivate disregard for their possible significance to normal CSF physiology. Eye movements are relentless in daily life, with saccades alone estimated to occur 180,000 times daily.^1^ If each saccade were conservatively estimated to shift retrobulbar perineural CSF in the SAS by only 5 µL, these 180,000 saccades would cumulatively change CSF volume in the retrobulbar SAS by 9 L/day. Whereas it is likely that most of the volume change during a sequence of eye movements would simply be to-and-fro movements like ocean waves breaking and receding from the seashore without overall directional bulk flow, small non-uniformities in rate of volume change and non-uniform flow resistance due to arachnoid trabeculations in the SAS could drive bulk CSF flow, analogous to the way waves on the seashore can sometimes carry away sand or objects. Hypothetically, a 1% difference in inward versus outward directional asymmetry of flow in the SAS could produce 90 mL bulk CSF flow in the SAS during the course of 1 day. A pumping mechanism driven by eye movements could thus be extremely inefficient, yet sufficiently effective to circulate CSF between the eyes and brain.

### Implications for Glaucoma

The current study has implications for the pathophysiology of POAG with normal IOP. If circulation of the retrobulbar CSF contributes to nutrition of the intraorbital ON or to removal of its waste products as has been proposed,^12^ then these functions may be impaired by the approximately 30% reduction in SAS volume change during adduction demonstrated here for POAG without abnormally elevated IOP. However, SAS volume changes in abduction and vertical ductions appear normal for these optic neuropathies.

It has been proposed that ON tethering during numerous cycles of adduction in people whose ONs do not stretch, transmits force to the optic disc and peripapillary sclera and can damage the ON when the combined influences of ocular biomechanical properties are unfavorable.^16^^,^^27^^,^^28^ Although failure of the glaucomatous ON to stretch in adduction is evident from MRI data, it has not been certain if this stiffness might be the cause versus the effect of glaucomatous atrophy of the ON itself. The present paper now provides NA-AION as a comparison group similar to POAG in that both exhibit a similar degree of ON atrophy representing about 10% volume reduction from healthy controls (see Fig. 5). Despite this similarity, NA-AION exhibits a small but significant ON elongation in adduction (see Fig. 7) without globe retraction (see Fig. 8), despite the overall greater ON redundancy in NA-AION relative to both POAG, and control subjects in both adduction and abduction (see Fig. 6). This greater ON path redundancy is likely attributable to the simple geometry of shorter axial length in the relatively hyperopic eyes with crowded optic discs that are susceptible to NA-AION. In the current study, axial length was about 1.5 mm shorter in NA-AION than in POAG, which requires the junction of the ON with the globe to shift less distance for the same angle of adduction. It also appears that ON length does not necessarily shorten to the minimum sufficient to permit free globe rotation. Redundancy of the ON path was greater than the healthy controls in both affected and unaffected eyes in monocular NA-AION.

### Implications for Pathogenesis of NA-AION

The current findings may implicate eye movements in the pathogenesis of NA-AION, but not by ON tethering. As seen in Figure 6, ON path redundancy in NA-AION is greater than normal, suggesting that neither horizontal nor vertical eye movements produce abnormal ON tethering. In NA-AION, the ON does elongate significantly in adduction, although less than normal (see Fig. 7B). The globe does not retract in large angle adduction (see Fig. 8A), where ON tethering does cause significant retraction.^16^^,^^28^ It remains possible, however, that eye movements that neither tether the ON nor retract the globe may nevertheless locally deform the optic disc and peripapillary tissues in a manner that could sometimes become pathological. This possibility might be evaluated by optical imaging, rather than MRI, of the effects of horizontal and vertical eye movements. If circulation of the retrobulbar CSF contributes to nutrition of intraorbital ON or to removal of its waste products, as has been proposed,^12^ then these functions may be impaired by the approximately 30% reduction in SAS volume change during adduction demonstrated here for NA-AION.

### Asymmetry

Consideration of the eight unilateral cases of NA-AION is informative. Aside from a statistically insignificant trend toward smaller ON partial volume in affected eyes that is presumably secondary to the acute pathologic event, none of the other parameters investigated here differed significantly between the two eyes. Sample size may be insufficient to detect associations related to laterality. Alternativeliy, an unstudied factor may be responsible for determining the laterality of the acute NA-AION.

### Papilledema

The current study did not include subjects with abnormally high intracranial pressure, which distends the ON sheath and causes swelling of the optic nerve head that is classically termed papilledema.^41^^,^^42^ This enlargement of the SAS can even indent the posterior sclera. If normal eye movements normally drive the circulation of CSF in the retrobulbar SAS, it seems likely that the putative pumping mechanism would be impaired by a large increase in SAS volume relative to the size of the ON and amplitude of its excursions during eye movements. Such a defect in CSF pumping might amplify the pathologic changes caused by papilledema.

### Spaceflight-Associated Neuro-Ocular Syndrome 

A recently recognized form of optic disc swelling and enlargement of the ON sheath^43^ occurs during prolonged exposure to microgravity during space travel, and is termed spaceflight-associated neuro-ocular syndrome (SANS).^44^^,^^45^ Although the etiology of this condition remains mysterious, it has been proposed that SANS may result from sequestration or compartmentalization of CSF in the intraorbital SAS.^44^^–^^47^ Whereas initiation of the process may not be explained by the current suggestion that eye movement-driven ON shifts drive intraorbital CSF flow by pumping the SAS, it seems likely that such a putative pumping mechanism would lose effectiveness when the ON sheath becomes distended. This could augment and perpetuate the SAS distention once it begins in microgravity.

### Novel Optic Nerve Finding

This study also demonstrated a novel MRI finding in NA-AION. Two unilateral cases exhibited longitudinally extensive, bright signal on T2 imaging in the deep orbit of the involved eye. Prior studies have demonstrated occasional, generally patchy abnormalities on diffusion-weighted and post-contrast imaging most commonly affecting the optic disc and occasionally the intraorbital ON,^48^ but, to our knowledge, T2 abnormalities have not previously been observed. This finding is, however, similar in both prevalence and location to the ON abnormality reported by Rizzo et al. for short T1 inversion recovery imaging in about 15% of patients with NA-AION,^49^ and resembles the T2 abnormality recently reported in glaucoma.^50^ It appears that this T2 signal reflects prolonged and extensive abnormality in the intraorbital ON, which would not be surprising even if the inciting ischemic event were at or near the optic disc.

### Globe Translation

Whereas medial translation in adduction and lateral translation in abduction have been previously reported for healthy subjects,^51^^–^^53^ patients with horizontal strabismus,^54^ and subjects with POAG,^16^^,^^28^ the current study extends these findings to NA-AION, albeit with modestly greater translation in abduction in NA-AION than in the additional group of control subjects (see Fig. 8B). Translation during vertical duction has received less attention, although for 20 degree eccentricities close to those achieved in the current study, Moon et al. reported about 0.4 mm inferior translation during supraduction, and 0.4 mm superior translation during infraduction.^53^ The study of Moon et al. included high myopes with axial length as great as 29.5 mm, with 25.3 mm average axial length that was comparable to subjects with POAG in the current study (see Fig. 2). Although healthy subjects exhibited the translational trend during vertical duction reported by Moon et al. (see Fig. 8C), the translations did not significantly differ from zero, and the trend was absent in subjects with NA-AION. It appears that globe translation does not make as large a kinematic contribution to extraocular muscle leverage in vertical duction as is the case for horizontal duction, where it probably influences the quantitative effect of strabismus surgery.^51^

Limitations include the obvious small sample size of this exploratory sudy that only modestly exceeds that based on power computation for related but not identical phenomena. Nevertheless, most of the new phenomena were evident at a high level of statistical significance that would likely be maintained or enhanced with larger samples. It is worthwhile to appreciate that analysis of the dimensions of the intraorbital ON and SAS requires meticulous MRI technique including visual fixation for suppression of physiologic eye movements that can blur the images to make these structures indistinguishable, or limit their detection to only the most anterior image planes. The data set reported here may thus be enriched in cases featuring larger SAS volumes that were more feasible to measure, and less severe optic neuropathies that better maintained the capacity to support steady monocular visual fixation during MRI. If this is the case, the association of SAS volume and its gaze-related changes is likely to be stronger than reflected by the present quantitative data. The associations demonstrated here do not support determination of causality of optic neuropathy; such a determination would require additional evidence.

## Summary

Horizontal and vertical eye movements significantly alter CSF volume within the ON sheath in a manner that could serve to pump CSF in the SAS around the retrobulbar ON, and so contribute to ON health. This eye movement-related alteration in SAS volume is subnormal in POAG occurring at normal IOP, and in NA-AION, raising the possibility that the putative pumping mechanism may be related to the pathology of these optic neuropathies.
